# Heavy metal accumulation in the bark and leaves of *Juglans regia* planted in Artvin City, Turkey

**DOI:** 10.1080/13102818.2014.947076

**Published:** 2014-10-21

**Authors:** Yunus Dogan, Mehmet C. Unver, Ilker Ugulu, Mesude Calis, Nazmi Durkan

**Affiliations:** ^a^ Buca Faculty of Education, Dokuz Eylul University, Izmir, Turkey; ^b^ Faculty of Education, Artvin Coruh University, Artvin, Turkey; ^c^ Faculty of Education, Pamukkale University, Denizli, Turkey

**Keywords:** heavy metal, bark, leaf, *Juglans regia*, ICP–OES, Artvin

## Abstract

The aim of this study was to determine the level of heavy metals such as copper, iron, manganese, zinc, lead, nickel, cadmium and chromium concentrated in *Juglans regia* bark and leaf samples from different localities in Artvin, Turkey. Analysis of the heavy metals Cu, Fe, Mn, Zn, Pb, Ni, Cd and Cr in samples was carried out by inductively coupled plasma optical emission spectroscopy (ICP–OES; Perkin Elmer, Optima 8000 DV). Statistical significance was determined by analysis of variance (ANOVA). The comparisons were performed in order to determine whether there were any differences between *J. regia* bark and leaf samples in terms of average heavy metal accumulation levels. As a result of this study, the following mean concentrations were determined for *J. regia* bark samples: the contents of Cu, Fe, Mn, Zn, Pb, Ni, Cd and Cr (μg g^−1^, dry weight) ranged from 72.46 to 88.14, 14.40 to 628.0, 0.896 to 67.71, 7.000 to 28.52, 0.040 to 0.905, 1.031 to 2.744, 0.011 to 0.158 and 1.192 to 3.134, respectively. On the other hand, for *J. regia* leaf samples, the contents of Cu, Fe, Mn, Zn, Pb, Ni, Cd and Cr (μg g^−1^, dry weight) ranged from 0.339 to 13.80, 12.72 to 698.2, 1.001 to 204.6, 7.362 to 56.03, 0.158 to 0.665, 0.130 to 2.744, 0.041 to 0.114 and 0.508 to 2.767, respectively. In the statistical analysis, heavy metal accumulation values of *J. regia* bark and leaf samples for Cu, Ni and Cr were significantly different (*P* < 0.05).

## Introduction

Heavy metal pollution is known to be responsible for serious environmental problems and risks to humans, including decreased soil microbial activity, fertility and yield losses.[1] Actually, large areas of land are contaminated with heavy metals originating from urban activities (municipal sewage sludge and waste incinerators), agricultural practices (fertilizer and pesticide application), and industrial processing (metalliferous mining, the smelting industry, printing factories and tanneries).[2] In contrast to organic pollutants, heavy metals are not biodegradable, since they have the ability to accumulate in organisms. As a result, there is increased attention on the use of plant parts such as leaves, shoots and bark for biomonitoring.[3–6]

Biomonitoring may be defined as the use of organisms or biomaterials to obtain information on certain compounds in the biosphere.[7] The main advantage of using biomonitors for environmental surveillance is their lower cost compared with direct methods of pollution measurement, since no collecting or measuring equipment has to be installed and protected against vandalism.[8,9] If biomonitors are distributed in a wide enough area and occur frequently enough, they can be used over large areas for recording and evaluating heavy metal accumulations. Furthermore, they make it possible to identify the sources of emissions and verify the overland transportation of heavy metals.[8–14]

Various researchers have explained the sources of heavy metal pollution. For example, Pb and Zn originate mainly from anthropogenic activities.[15–17] Major anthropogenic sources of Ni are burning of coal and oil, production of Cu, Ni and Pb, mining operations, steel works, and the cement industry.[18] Loppi et al. [19] have reported that plants are highly affected by contamination of the soil by Fe and Mn in the Mediterranean climate zone. However, some researchers have reported that airborne Mn originates mainly from the soil.[17,20] Fe originates both from anthropogenic and natural sources.[17]

The elements Fe, Zn, Mn and Ni are considered to be micronutrients that are essential for plant growth.[21,22] Enrichments of mostly lithogenic Ni and Zn in the top soil and corresponding depletions in the subsoil are often observed and explained as the result of nutrient cycling.[23] Mn occurs in soil mainly in the form of compounds of Mn^2+^ and as Mn oxide.[13] Guevera et al. [24] reported a strong correlation between elements abundant in the soil and elements existing in plants. The elements in plants might stem from the soil.

The aim of this study was: (1) to determine the Cu, Fe, Mn, Zn, Pb, Ni, Cd and Cr contents in *Juglans regia* L. (Juglandaceae) bark and leaf samples from different localities in Artvin, Turkey; and (2) to compare the accumulation levels among bark and leaf samples of *J. regia*. For these purposes, plant samples were analysed to determine heavy metal contents by using inductively coupled plasma optical emission spectroscopy (ICP–OES).

## Materials and methods

### General characteristics and economical importance of the species

*Juglans regia* L. is a plant that is naturally distributed in a wide area encompassing countries south of the Carpathian Mountains, Eastern Europe, Turkey, Iraq and Eastern Iran to Himalayan Mountains.[9] Turkey is one of the native countries of *J. regia* and occupies the third place in the world following China and USA in terms of walnut production. With a production of 177,000 tonnes, Turkey provides 7.93% of the worldwide walnut production.[25] Walnut is widely consumed in Turkey. In addition to that, tree bark, fruit shells and husks, and walnut leaves are commonly used in pharmaceutical and cosmetic industries and as dyestuff in carpet and textile industries.[26–27]

Walnut is a species that can adapt to various climate conditions. It is grown economically in areas up to 1700 m above sea level (a.s.l.). In addition, it is possible to come across walnut trees bearing fruit in the Mediterranean coastline of Turkey.[28] However, under these climate conditions, i.e. extreme summer heat, fruit peel and leaves can burn and inner walnut fruit can shrink.[25]

### Study area

**Figure 1.**
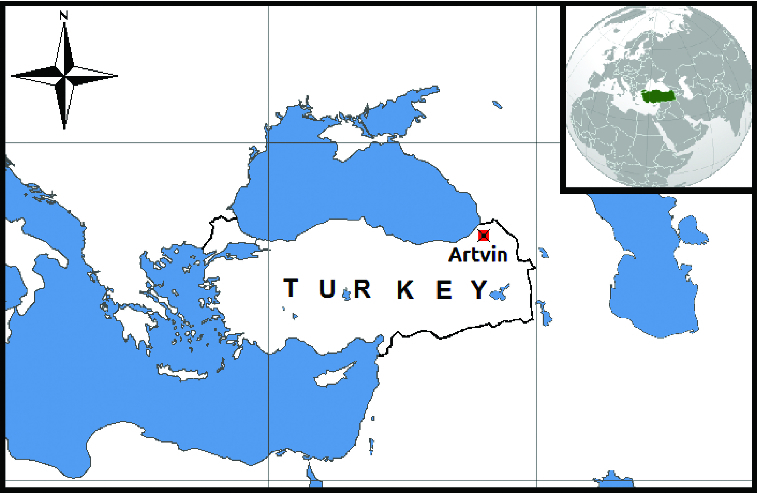
Map of Turkey and Artvin town location in the country.

Artvin is a city in the Black Sea region with an area of 7367 km^2^, situated between 40° 35′ and 41° 32′ northern latitudes and 41° 07′ and 42° 00′ eastern longitudes. The city area corresponds to 0.9% of the land area of Turkey, which is 783,577 km^2^. Its neighbours are Ardahan to the east, Erzurum to the south, Rize to the west, Georgia to the north and the Black Sea to the northwest (Figure 1). It has a shoreline of 34 km and the altitude of Artvin city centre is 240 m a.s.l.

In terms of climate, Artvin is the most capricious city in the Eastern Black Sea region. The area encompassing the coast and Cankurtaran Mountain range displays a typical wet Black Sea climate in all seasons. The climate of the area from the Cankurtaran Mountain range to Borcka and Artvin city centre displays a Black Sea climate with colder winters and less rain. The climate of Ardanuc and Yusufeli is a mixture of part Continental climate and Mediterranean climate with hot and dry summers, and warm and less wet winters compared with Continental climate. This diversity in climate is reflected in the plant diversity in the area. Artvin is among the important cities in Turkey in terms of plant diversity and endemic species. There are 1268 identified plant species and 119 of them are endemic.

Walnut bark and leaf samples used in the study were collected from 10 different localities in Artvin city centre. Special attention was paid to collect the samples from areas as varied as possible. The first station where the samples were collected was Hastane (Hospital); the second station was Ogretmen Evi (Teacher House); the third station was Valilik (Governorship); the fourth station was Eski Otogar (Early terminal); the fifth station was Hapishane (Prison); the sixth station was Adliye (Court House); the seventh station was DSI–1 (Water Management Office); the eighth station was Ustgecit (Footbridge); the ninth station was Sehir Girisi (Town entry); and the tenth station was Dere Mahallesi (Dere district).

### Study materials

Samples were collected from different localities of the research area during the autumn of 2012. One bark and one leaf sample were collected from each sampling station. During the process of collection, *J. regia* samples were assigned a number in the field notebook and the names of the localities were noted. The recorded samples were collected and placed in polyethylene bags with corresponding numbers. All the polyethylene bags were washed with 5% nitric acid and distilled water and dried at room temperature before the experiments.

### Analytical process

The collected plant samples were dried at 50 °C for 48 hours. The dried samples were kept in polyethylene bags until analysis. After drying, 25 mL of nitric acid was added to 2 g of dried sample. It was heated slowly in a heater for 30 minutes and left to cool. Then, 15 mL of perchloric acid was added and boiled for about 1 hour in a magnetic heater until it became colourless. After cooling, 50 mL of deionized water was added. The samples were kept in polyethylene bottles in a fridge at 4 °C until analysis.[5] Teflon wares and suprapure (Merck) chemicals were used in the analyses.

The amounts of copper, iron, manganese, zinc, lead, nickel, cadmium and chromium were measured from *J. regia* samples collected from the different localities in Artvin. Analysis of the elements in the plants was carried out by ICP–OES (Perkin Elmer, Optima 8000 DV).

### Statistical analysis

Statistical significance was determined by analysis of variance (ANOVA). The ANOVA comparisons were made in order to determine whether there were any differences between the means of the bark and leaf samples of *J. regia* in terms of Cu, Fe, Mn, Zn, Pb, Ni, Cd and Cr accumulation levels. Differences at *P* < 0.05 were considered to be significant. The Statistical Package for Social Sciences (SPSS) was used in the ANOVA for the data collected.

## Results and discussion

Accumulation levels of Cu, Fe, Mn, Zn, Pb, Ni, Cd and Cr (μg g^−1^, dry weight) in *J. regia* bark and leaf samples collected from different localities in Artvin are shown in Tables 1 and 2. The concentrations of elements were determined by ICP–OES. As a result of this study, the following mean concentrations were determined for the *J. regia* bark samples: the contents of Cu, Fe, Mn, Zn, Pb, Ni, Cd and Cr (μg g^−1^, dry weight) ranged from 72.46 to 88.14, 14.40 to 628.0, 0.896 to 67.71, 7.000 to 28.52, 0.040 to 0.905, 1.031 to 2.744, 0.011 to 0.158 and 1.192 to 3.134, respectively (Table 1). On the other hand, for the *J. regia* leaf samples, the contents of Cu, Fe, Mn, Zn, Pb, Ni, Cd and Cr (μg g^−1^, dry weight) ranged from 0.339 to 13.80, 12.72 to 698.2, 1.001 to 204.6, 7.362 to 56.03, 0.158 to 0.665, 0.130 to 2.744, 0.041 to 0.114 and 0.508 to 2.767, respectively (Table 2).

**Table 1.** Heavy metal accumulation values in walnut bark samples (μg g^−1^, dry weight).

Sample	Cu	Fe	Mn	Zn	Pb	Ni	Cd	Cr
1	75.71	168.5	62.65	28.52	0.309	2.129	0.064	1.204
2	76.38	444.9	54.04	7.00	0.362	2.744	0.058	3.134
3	80.32	539.5	31.68	12.51	0.152	2.040	0.096	1.907
4	88.14	14.40	0.896	11.45	0.905	2.396	0.098	2.748
5	78.34	358.3	26.06	11.19	0.188	2.186	0.055	2.497
6	79.24	410.3	22.60	26.60	0.263	1.691	0.049	1.870
7	75.42	327.1	60.67	7.407	0.040	2.190	0.079	2.206
8	76.85	628.0	37.92	18.97	0.211	2.432	0.022	2.699
9	72.46	219.5	45.71	9.892	0.176	1.031	0.011	1.192
10	78.07	417.6	67.71	11.02	0.041	2.422	0.158	2.766
Min.	72.46	14.40	0.896	7.000	0.040	1.031	0.011	1.192
Max.	88.14	628.0	67.71	28.52	0.905	2.744	0.158	3.134
Mean	78.1 ± 1.31	353 ± 57.1	40.9±6.68	14.4 ± 2.41	0.26 ± 0.07	2.12 ± 0.15	0.06 ± 0.01	2.22 ± 0.21

**Table 2.** Heavy metal accumulation values in walnut leaf samples (μg g^−1^, dry weight).

Sample	Cu	Fe	Mn	Zn	Pb	Ni	Cd	Cr
1	4.786	519.0	112.9	56.03	0.665	2.744	0.079	2.767
2	8.693	281.1	73.55	16.70	0.262	0.511	0.076	0.569
3	8.203	397.9	26.95	20.05	0.333	0.432	0.073	0.735
4	9.503	332.5	204.6	7.362	0.158	0.130	0.041	0.508
5	13.80	12.72	1.001	7.829	0.381	0.635	0.074	0.770
6	3.360	450.6	21.35	34.56	0.470	0.629	0.065	0.791
7	0.383	452.0	42.47	28.10	0.510	0.886	0.081	0.897
8	0.339	651.0	46.36	42.10	0.240	0.961	0.049	1.073
9	3.815	698.2	50.20	35.22	0.430	0.723	0.056	2.056
10	7.745	375.9	124.8	13.90	0.319	0.860	0.114	0.709
Min.	0.339	12.72	1.001	7.362	0.158	0.130	0.041	0.508
Max.	13.80	698.2	204.6	56.03	0.665	2.744	0.114	2.767
Mean	6.06±1.35	417±61.2	70.4±19.3	26.1±5.02	0.37±0.04	0.85±0.22	0.07±0.02	1.08±0.23

Due to the fact that the accumulation of pollutants varies from plant to plant and also among various parts of a plant species,[13,29,30] proper selection of plant species for biomonitoring plays an important role in the determination of the extent of toxic metal pollution in the environmental media such as soil, water and air.[31] In order to overcome these problems, enlarged attention is being paid to the use of plant parts such as the leaf, shoot and bark for biomonitoring.[9,13,14,20,29,32]

In related literature, there are many studies focusing on using the bark and needles of the plant species as a biomonitor. Some of them are as follows: Galuszka [33] used the needles and bark of the Scots pine (*Pinus sylvestris*) with some other species, Dmuchowski and Bytnerowicz,[34] Yilmaz and Zengin,[35] used the needles of the Scots pine, Ceburnis and Steinnes [26] used the needles of the Norway spruce (*Picea abies*) and juniper (*Juniperus communis*), Baslar et al. [11] used the leaves of poplar (*Populus nigra*), Pyatt [36] used the needles of the Corsican pines (*Pinus nigra* subsp. *laricio*), and Baslar et al. [11] used the needles of the Turkish red pine (*Pinus brutia*) to monitor heavy metal pollution. On the other hand, some other researchers, such as Huhn et al.,[37] Poikolainen,[38] Schulz et al.,[39] Pacheco et al.,[40] and Harju et al.,[41] studied the Scots pine bark; Takala et al.,[42] Loppi et al.,[43] and Van Dobben et al. [44] used the pine bark and lichens living on the bark; and Lippo et al. [45] and Samecka–Cymerman at al. [32] used the pine bark and mosses living on them. However, in the aforementioned studies, the bark and leaf samples were evaluated independently and the question of which part is more useful in terms of biomonitoring was left unanswered. The aim of present study was to determine the pollution levels of Cu, Fe, Mn, Zn, Pb, Ni, Cd and Cr by using the bark and the leaves of the *J. regia* as a bioindicator in Artvin City of Turkey and to compare the accumulation levels of the bark and leaves of the walnut samples collected from different localities in the study area. Results of the study demonstrated that the bark and the leaves of the *J. regia* can be used as effective biomonitors for detecting heavy metal pollution in the study area. However, while the heavy metal accumulation in the bark samples was observed to be higher for Cu, Ni, and Cr, for the other heavy metals (Fe, Mn, Zn, Pb and Cd), accumulation values were found to be higher in leaf samples.

Normal natural concentration ranges of heavy metals for land plants have been reported as Cd: 0.2–2.4 μg g^−1^, Cu: 2–20 μg g^−1^, Ni: 1–5 μg g^−1^, Fe: 70–700 μg g^−1^, Pb: 1–13 μg g^−1^, Mn: 20–700 μg g^−1^ and Zn: 20–400 μg g^−1^.[17,46] Comparison of our results with these findings showed that in terms of heavy metal accumulation, Cu accumulation above pollution levels was observed only in bark samples of *J. regia*. It is remarkable that among the eight heavy metals that were investigated, only Cu displayed high amounts of accumulation in all bark samples.

In nature, Cu occurs in rocks, water and air, and is essential for the normal growth and metabolism of all living organisms.[47–49] It is present naturally in the environment in the elemental form, but most commercial production is from sulphides and oxide minerals.[50] Cu is still widely used in the manufacturing of electrical equipments; in construction, such as roofing and plumbing; and industrial machinery, such as heat exchangers and alloys. Cu also has a wide range of other applications in agriculture (pesticides and fungicides), wood preservation.[21,47] It is thought that in order to determine and prevent Cu accumulation in the area, it would be beneficial to consider the reasons for high Cu accumulation in this perspective. When the activities about Cu in the study area were investigated it was seen that mining industry and activities related to Cu were reasonably expanded. Moreover, these activities are widespread throughout the Eastern Black Sea Region. For this reason, additional studies on heavy metal accumulation in this region would be beneficial to determine heavy metal pollution like Cu.

Detailed literature reviews showed that some researchers have performed the studies related to heavy metal accumulation in mosses and mushrooms in Artvin and its environs.[17,45,51] However, any study on heavy metal accumulation in vascular plants was not found. This study aims to determine heavy metal accumulation in *J. regia* as a vascular plant and the study with this feature is the first in the city. However, Kucukbay and Kuyumcu [52] used leaves of *Thymus kotschyanus* from Erzurum, a neighbouring city of Artvin, for their study where they examined heavy metal contents in *Thymus* species as a vascular plant. In the study, they determined mean values of 10.1 μg g^−1^ for Cu, 849.0 μg g^−1^ for Fe, 52.0 μg g^−1^ for Mn, 36.2 μg g^−1^ for Zn, 0.7 μg g^−1^ for Pb and 0.08 μg g^−1^ for Cd. No accumulation of Ni was detected. When the results of this study and those of Kucukbay and Kuyumcu [52] were compared, it was seen that more accumulation was present by Kucukbay and Kuyumcu [52] in all heavy metals except Cu.

When mean accumulation values in bark and leaf samples of *J. regia* were compared, it was seen that accumulation values of Cu, Ni and Cr were higher in bark samples, and higher for other elements in leaf samples (Tables 1 and 2). The ANOVA test was conducted in order to determine whether this difference between bark and leaf samples was statistically significant (Table 3). In the statistical analysis, comparison of heavy metal accumulation values of *J. regia* bark and leaf samples for Cu, Ni and Cr found a significant difference (*P* < 0.05), while there was no significant difference for Fe, Mn, Zn, Pb and Cd. Considering that bark has a longer lifespan than leaves in the growth process of a plant, it could be expected that heavy metal accumulation values in bark will be higher than those in leaves. However, when the findings of this study were evaluated, it was seen that this was the case only in three elements (Cu, Ni and Cr) out of the eight heavy metals analysed. For the other heavy metals (Fe, Mn, Zn, Pb and Cd), accumulation values were found to be higher in leaf samples. However, statistical analyses support the assumption of higher accumulation values in bark samples by revealing that only high accumulation values of Cu, Ni and Cr in bark were statistically significant.

**Table 3.** ANOVA analysis of heavy metal accumulation values in *J. regia* planted in Artvin, Turkey.

		df	*F*	Sig.	
Cu	Between groups		1	1453.09	.000
	Within groups		18		
	Total		19		
					
Fe	Between groups		1	.589	.453
	Within groups		18		
	Total		19		
					
Mn	Between groups		1	2.068	.168
	Within groups		18		
	Total		19		
					
Zn	Between groups		1	4.428	.050
	Within groups		18		
	Total		19		
					
Pb	Between groups		1	1.511	.235
	Within groups		18		
	Total		19		
					
Ni	Between groups		1	22.304	.000
	Within groups		18		
	Total		19		
					
Cd	Between groups		1	.015	.904
	Within groups		18		
	Total		19		
					
Cr	Between groups		1	13.046	.002
	Within groups		18		
	Total		19		

## Conclusion

Ethnobotanical studies clearly show that walnut leaves were traditionally used in the past and nowadays internally or externally for medicinal purposes, which makes accumulation values in the plant important in terms of human health.[12,15,53–59] Consequently, it is vital to consume walnuts in this perspective, as is the case in the consumption of other food. The results of this study show that the area is not problematic in terms of heavy metal accumulation. However, it is possible to say that there is pollution with Cu. New studies performed from this point of view will be beneficial in terms of determining heavy metal accumulation values in the area.

## References

[cit0001] McGrath SP, Chaudri AM, Giller KE (1995). Longterm effects of metals in sewage sludge on soils, microorganisms and plants. J Ind Microbiol Biotechnol..

[cit0002] Lasat MM (2002). Phytoextraction of toxic metals: a review of biological mechanisms. J Environ Qual..

[cit0003] Baslar S, Kula I, Dogan Y, Yildiz D, Ay G (2009). A study of trace element contents in plants growing at Honaz Dagi–Denizli, Turkey. Ekoloji..

[cit0004] Dogan Y, Ugulu I, Baslar S. (2010). Turkish red pine as a biomonitor: a Comparative study of the accumulation of trace elements in needles and barks. Ekoloji.

[cit0005] Durkan N, Ugulu I, Unver MC, Dogan Y, Baslar S (2011). Concentrations of trace elements aluminum, boron, cobalt and tin in various wild edible mushroom species from Buyuk Menderes River Basin of Turkey by ICP–OES. Trace Elem Electrolytes..

[cit0006] Ugulu I, Dogan Y, Baslar S, Varol O (2012). Biomonitoring of trace element accumulation in plants growing at Murat Mountain. Int J Environ Sci Technol..

[cit0007] Markert B, Breure T, Zechmeister H (2003). Bioindicators and biomonitors – principles, concepts and applications.

[cit0008] Mertens J, Luyssaert S, Verheyen K (2005). Use and abuse of trace metal concentrations in plant tissue for biomonitoring and phytoextraction. Environ Pollut..

[cit0009] Smodis B, Pignata ML, Saiki M (2004). Validation and application of plants as biomonitors of trace element atmospheric pollution – a co-ordinated effort in 14 countries. J Atmospheric Chem..

[cit0010] Baslar S, Dogan Y, Bag H, Elci A (2003). Trace element biomonitoring by needles of *Pinus brutia* from Western Anatolia. Fresenius Environ Bull..

[cit0011] Baslar S, Dogan Y, Yenil N, Karagoz S, Bag H (2005). Trace element biomonitoring by leaves of *Populus nigra* L. from Western Anatolia, Turkey. J Environ Biol..

[cit0012] Dogan Y, Durkan N, Baslar S (2007). Trace element pollution biomonitoring using the bark of *Pinus brutia* (Turkish red pine) in the Western Anatolian part of Turkey. Trace Elem Electrolytes.

[cit0013] Singh M, Goel P, Singh A (2005). Biomonitoring of lead in atmospheric environment of an urban center of the Ganga Plain, India. Environ Monit Assess..

[cit0014] Tomasevic M, Rajsic S, Dordevic D, Tasic M, Krstic J, Novakovic V (2004). Heavy metals accumulation in tree leaves from urban areas. Environ Chem Lett..

[cit0015] Alfani A, Baldantoni D, Maisto G, Bartoli A, Virzo De Santo A (2000). Temporal and spatial variation in C, N, S and element contents in the leaves of *Quercus ilex* within the urban area of Naples. Environ Pollut..

[cit0016] Blok J (2005). Environmental exposure of road borders to zinc. Sci Total Environ..

[cit0017] Oliva SR, Rautio P (2005). Spatiotemporal patterns in foliar element concentrations in *Ficus microcarpa* L. f. growing in an urban area: implications for biomonitoring studies. Ecol Indicators..

[cit0018] Nriagu JO, Pacyna J (1988). Quantitative assessment of worldwide contamination of air, water and soils by trace metals. Nature..

[cit0019] Loppi S, Giomerelli B, Bargagli R (1999). Lichens and mosses as biomonitors of trace elements in a geothermal area (Mt. Amiata, Central Italy). Cryptog Mycolog..

[cit0020] Bargagli R (1998). Plants as biomonitors. Trace elements in terrestrial plants: an ecophysiological approach to biomonitoring and biorecovery.

[cit0021] Reid RJ (2001). Mechanisms of micronutrient uptake in plants. Aust J Plant Physiol..

[cit0022] Stoponenicne L, Tautkus S, Kazlauskas R (2003). Determination of zinc in plants and grains by atomic absorbsion spectrometry. Chemijia..

[cit0023] Luster J, Zimmermann S, Frey B, Brunner I, Luscher P, Walthert L, Blaser P (2006). Schwermetalle in Schweizer Waldböden. Wald und Holz.

[cit0024] Guevera SR, Arribere MA, Calevela S, Roman RG (1995). Elemental composition of lichens at Nahuel Huapi National Park, Patagonia, Argentina. J Radioanal Nucl Chem.

[cit0025] Bayazit S. (2011). Bazi ceviz (*Juglans regia* L.) genotiplerinin Yayladagi (Hatay) kosullarındaki fenolojik ozellikleri ve yan dal verimliligi. Ataturk Univ Ziraat Fak Derg..

[cit0026] Ceburnis D, Steinnes E (2000). Conifer needles as biomonitors of atmospheric heavy metal deposition: comparison with mosses and precipitation, role of the canopy. Atmospheric Environ..

[cit0027] Yigit D, Yigit N, Aktas E, Ozgen U (2009). Antimicrobial activity of walnut (*Juglans regia* L.). Turk Mikro Cem Der..

[cit0028] Akca Y (2001). Ceviz Yetistiriciligi.

[cit0029] Baycu G, Tolunay D, Ozden H, Gunebakan S (2006). Ecophysiological and seasonal variations in Cd, Pb, Zn and Ni concentrations in the leaves of urban deciduous trees in Istanbul. Environ Pollut..

[cit0030] Huseyinova R, Kutbay HG, Bilgin A, Kilic D, Horuz A, Kirmanoglu C (2009). Sulphur and some heavy metal contents in foliage of *Corylus avellana* and some roadside native plants in Ordu province, Turkey. Ekoloji..

[cit0031] Kaya G, Yaman M (2008). Trace Metal Concentrations in Cupressaceae leaves as biomonitors of environmental pollution. Trace Elem Electrolytes..

[cit0032] Samecka–Cymerman A, Stankiewicz A, Kolon K, Kempers AJ (2009). Self-organizing feature map (neural networks) as a tool to select the best indicator of road traffic pollution (soil, leaves or bark of *Robinia pseudoacacia* L.). Environ Pollut..

[cit0033] Galuszka A (2005). The chemistry of soils, rocks and plant bioindicators in three ecosystems of the Holy Cross Mountains, Poland. Environ Monit Assess..

[cit0034] Dmuchowski W, Bytnerowicz A (1995). Monitoring environmental pollution in Poland by chemical analysis of Scots pine (*Pinus sylvestris* L.) needles. Environ Pollut..

[cit0035] Yilmaz S, Zengin M (2004). Monitoring environmental pollution in Erzurum by chemical analysis of Scots pine (*Pinus sylvestris* L.) needles. Environ Int..

[cit0036] Pyatt FB. (1999). Comparison of foliar and stem bioaccumulation of heavy metals by Corsican pines in the Mount Olympus Area of Cyprus. Ecotox Environ Safe..

[cit0037] Huhn G, Schulz G, Stark HJ, Tolle R, Schuurmann G (1995). Evaluation of regional heavy metal deposition by multivariate analysis of element contents in pine tree barks. Water Air Soil Pollut..

[cit0038] Poikolainen J. (1997). Sulphur and heavy metal concentrations in Scots pine bark in northern Finland and the Kola Peninsula. Water Air Soil Pollut..

[cit0039] Schulz H, Popp P, Huhn G, Stark HJ, Schuurmann G (1999). Biomonitoring of airborne inorganic and organic pollutants by means of pine tree barks. Sci Total Environ..

[cit0040] Pacheco AMG, Freitas MC, Barros LIC, Figueira R (2001). Investigating tree bark as an air–pollution biomonitor by means of neutron activation analysis. J Radioanal Nucl Chem..

[cit0041] Harju L, Saarela KE, Rajander J, Lill JO, Lindroos A, Heselius SJ (2002). Preconcentration of trace elements in biological materials by dry ashing for TTPIXE–analyses. Nucl Instruments Meth B..

[cit0042] Takala K, Olkkonen H, Salminen R (1994). Determination of zinc in plants and grains by atomic absorbsion spectrometry. Environ Pollut..

[cit0043] Loppi S, Nelli L, Ancora S, Bargagli R (1997). Passive monitoring of trace elements by means of tree leaves, epiphytic lichens and bark substrate. Environ Monit Assess..

[cit0044] Van Dobben HF, Wolterbeek HTH, Wamelink GWW, Ter Braak CJF (2001). Relationship between epiphytic lichens, trace elements and gaseous atmospheric pollutants. Environ Pollut..

[cit0045] Lippo H, Poikolainen J, Kubin E (1995). The use of moss, lichen and pine bark in the nationwide monitoring of atmospheric heavy metal deposition in Finland. Water Air Soil Pollut..

[cit0046] Bowen HJM (1979). Environmental chemistry of the elements.

[cit0047] Kanoun–Boule M, De Albuquerque MB, Nabais C, Fretias H, Prasad MNV (2008). Copper as an environmental contaminant: phytotoxicity and human health implications. Trace elements as contaminants and nutrients: consequences in ecosystems and human health.

[cit0048] Yildiz D, Kula I, Ay G, Baslar S, Dogan Y (2010). Determination of trace elements in plants of Mt. Bozdag, Izmir, Turkey. Arch Biol Sci..

[cit0049] Kula I, Yildiz D, Dogan Y, Ay G, Baslar S (2010). Trace element contents in plants growing at Mt. Akdag, Denizli. Biotechnol Biotechnol Equipments..

[cit0050] Georgopoulos PG, Roy A, Yonone-Lioy MJ, Opiekun RE, Lioy PJ (2001). Environmental copper: its dynamics and human exposure issues. J Toxicol Environ Health B Crit Rev..

[cit0051] Koz B, Cevik U, Akbulut S (2012). Heavy metal analysis around Murgul (Artvin) copper mining area of Turkey using moss and soil. Ecol Indicators..

[cit0052] Kucukbay FZ, Kuyumcu E (2010). Determination of trace element contents of *Thymus* species from Turkey. Turkish J Chem..

[cit0053] Dogan Y, Baslar S, Ay G, Mert HH (2004). The use of wild edible plants in Western and Central Anatolia (Turkey). Econ Bot.

[cit0054] Dogan Y, Ugulu I, Durkan N (2013). Wild edible plants sold in the local markets of Izmir. Pakistan J Bot..

[cit0055] Dogan Y, Ugulu I (2013). Medicinal plants used for gastrointestinal orders in some districts of Izmir Province, Turkey. Stud Ethno-Med..

[cit0056] Ugulu I, Baslar S, Yorek N, Dogan Y (2009). The investigation and quantitative ethnobotanical evaluation of medicinal plants used around Izmir province, Turkey. J Med Plant Res..

[cit0057] Dogan Y, Baslar S, Mert HH, Ay G (2003). Plants used as natural dye sources in Turkey. Econ Bot..

[cit0058] Ugulu I, Baslar S (2010). The determination and fidelity level of medicinal plants used to make traditional Turkish salves. J Altern Complement Med..

[cit0059] Ugulu I (2012). Fidelity level and knowledge of medicinal plants used to make therapeutic Turkish baths. Stud Ethno-Med..

